# Deterministically Entangling Two Remote Atomic Ensembles via Light-Atom Mixed Entanglement Swapping

**DOI:** 10.1038/srep25715

**Published:** 2016-05-11

**Authors:** Yanhong Liu, Zhihui Yan, Xiaojun Jia, Changde Xie

**Affiliations:** 1 State Key Laboratory of Quantum Optics and Quantum Optics Devices, Institute of Opto-Electronics, Shanxi University, Taiyuan, 030006, People’s Republic of China; 2 Collaborative Innovation Center of Extreme Optics, Shanxi University, Taiyuan, 030006, People’s Republic of China

## Abstract

Entanglement of two distant macroscopic objects is a key element for implementing large-scale quantum networks consisting of quantum channels and quantum nodes. Entanglement swapping can entangle two spatially separated quantum systems without direct interaction. Here we propose a scheme of deterministically entangling two remote atomic ensembles via continuous-variable entanglement swapping between two independent quantum systems involving light and atoms. Each of two stationary atomic ensembles placed at two remote nodes in a quantum network is prepared to a mixed entangled state of light and atoms respectively. Then, the entanglement swapping is unconditionally implemented between the two prepared quantum systems by means of the balanced homodyne detection of light and the feedback of the measured results. Finally, the established entanglement between two macroscopic atomic ensembles is verified by the inseparability criterion of correlation variances between two anti-Stokes optical beams respectively coming from the two atomic ensembles.

It is crucial to establish entanglement between remote nodes with stationary quantum systems in a quantum network^1^. The discrete-variable (DV) entanglement of single photons and the continuous-variable (CV) entanglement of optical modes have been deeply studied^2^,^3^ and applied in a variety of quantum information protocols, such as quantum teleportation, quantum entanglement swapping, quantum secret sharing, quantum computing, and so on^4^,^5^,^6^,^7^,^8^,^9^,^10^,^11^,^12^. Entanglement swapping can entangle two quantum systems that have never directly interacted with each other, and thus is a significant protocol in quantum communication^6^,^7^,^8^. With the development of quantum information, quantum network consisting of quantum channels and quantum nodes have attracted more and more attentions. Light is the best quantum information carrier and is used as quantum channels in quantum networks, usually. Meanwhile atomic ensembles are one of the promising candidates for quantum nodes to process and memory quantum information^1^. The entanglement of light and atoms is utilized to transfer quantum information between different quantum systems. Besides cavity quantum electromagnetic dynamics system in which the interaction of light and atoms is enhanced by optical cavity, atomic ensembles are the proper quantum nodes as a result of high optical density^13^,^14^. The Spontaneous Raman Scattering (SRS) process has been used to generate DV entanglement between single photons and atoms^15^,^16^,^17^. The CV entanglement of light and atomic ensembles has also been obtained by means of quantum non-demolition (QND) interaction^18^,^19^. The schemes of producing CV entanglement between light and atoms via three-wave or four-wave mixing have been proposed^20^,^21^. With the help of entanglement of light and atoms, the teleportation from photonic quantum bits and optical quantum modes to atomic spin wave states have been experimentally achieved, respectively^18^,^22^. The information transfer from one atomic node to another node has been realized as well^19^,^23^.

In practical applications of quantum information, the inevitable transmission loss limits the communication distance. Briegel H. J. *et al.*^24^ have introduced the concept of quantum repeater to overcome this problem^24^. Quantum nodes play the role of quantum repeater, and the entanglement among different nodes has to be required for constructing large-scale quantum networks and transferring quantum states. In DV regime atom-atom entanglement has been realized by mapping entangled photons into two sets of trapped atomic ensembles^25^. Another approach of generating atom-atom entanglement is DLCZ (Duan, Lukin, Cirac and Zoller) scheme, which is based on entanglement between light and atoms as well as single photon probabilistic counting^26^,^27^. The atom-atom CV entanglement has been generated by means of QND interaction and dissipative mechanism^28^,^29^, respectively. However, all above-mentioned schemes of generating CV entanglement between atomic ensembles are realized in a local space and would not be suitable to build the entanglement between two remote nodes in quantum networks.

In this paper we propose a scheme to produce the deterministic entanglement between two remote atomic ensembles based on applying CV entanglement swapping between two mixed entangled systems of light and atoms. At first, two CV entangled states of light and atomic ensemble are respectively prepared via the SRS process. When a write pulse is applied on an atomic ensemble, the scattering Stokes light will be entangled with the atomic ensemble^20^. Second, two Stokes optical pulses respectively entangled with the two atomic ensembles are combined on an optical beam splitter and then detected by the balanced homodyne detector (BHD). Third, the detected correlation variances of amplitude and phase quadratures between the two Stokes optical beams are fedback to the spin wave state of one of the two atomic ensembles. In the case, the entanglement between two atomic ensembles is built by the quantum entanglement swapping. The theoretical analysis point out that the maximum entanglement can be obtained if the optimal gain factor is chosen. Finally, the entanglement between atomic ensembles is confirmed by mapping atomic spin wave states into the anti-Stokes optical states and measuring the correlation variances between two anti-Stokes optical states.

## Results

### Schematic of entanglement generation system

**Figure 1:**
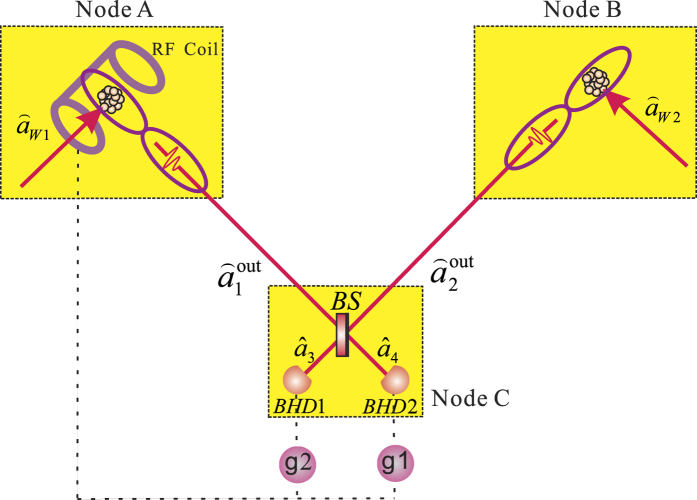
Schematic of atom-atom entanglement generation system. BS: beam splitter; BHD1 (2): balanced homodyne detector; RF Coil: radio frequency coil.

The schematic of the deterministic CV entanglement generation system between two remote atomic ensembles is shown in Fig. 1. The system involves two independent atomic ensembles A (B), a beam splitter (BS) and a pair of balanced homodyne detectors (BHD1, and BHD2). BHD1 (BHD2) composes of a beam splitter, a pair of photodiode detectors and a negative power combiner. BS, BHD1, and BHD2 are placed in a middle node C. Atomic ensemble A is put in the center of a radio frequency coil. Two Stokes optical pulses (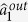 and 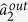) respectively produced by atomic ensembles A and B via SRS process are interfered on BS. The amplitude and phase quadratures of the output beams 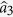 and 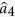 from BS are detected by BHD1 and BHD2, respectively. The interference of 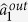 and 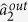 transfers the quantum information of the atomic ensembles A (B) to B (A) due to the existence of quantum entanglement between 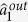 (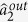) and atomic ensemble A (B). The signal detected by BHD1 and BHD2 are fed back to the atomic ensemble A via a classical channel, i.e. a radio frequency coil, to finally accomplish entanglement swapping and establish the entanglement between two atomic ensembles A and B^18^,^19^. The atomic spin state is related to magnetic fields generated by the feedback RF signal, with finite response time of the RF coil.

### Generation of the entanglement between light and atoms

**Figure 2:**
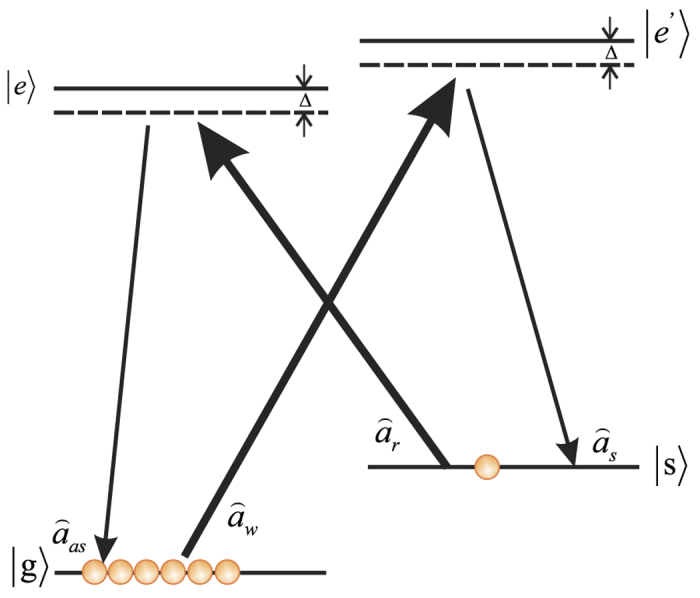
Atomic energy levels for the SRS. The atoms with a ground state |*g*〉, a meta-stable state |*s*〉, and two excited states |*e*〉 and |*e*’〉.

In quantum optical theory, optical fields are described by annihilation and creative operators 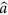 and 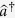, as well as the amplitude 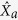 and the phase 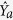 quadratures of light corresponding to the real and imaginary part of the annihilation operators 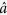, that is 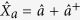, 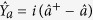. The collective atomic spin state 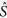 is described by the Stokes vector on Bloch sphere, which can be viewed as approximately satisfying the Bosonic field commutation relation 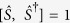
21. Similarly the amplitude 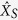 and the phase 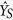 quadratures of atoms correspond to the real and imaginary part of the atomic spin wave operators 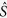, that is 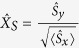, 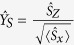. When the atomic spin wave is coupled with the optical signal and the pump fields via SRS process, the entanglement between light and atoms will be built^20^. The energy levels of atom asked for preparing the entangled state are shown in Fig. 2, which should have a ground state |*g*〉, a meta-stable state |*s*〉, and two excited states |*e*〉 and |*e*’〉. A lot of trapped atoms have such energy configuration, for example, ^87^*Rb* atoms. The Stokes optical field 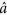 and the collective atomic spin wave 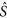 are simultaneously generated under the controlled interaction of the write pulse 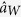. The effective interaction Hamiltonian of the system in the interaction picture is written as:


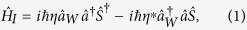


where the interaction constant of light and atoms 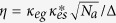, *κ*_*eg*_, *κ*_*es*_ are the coupling coefficients between light and atoms, *N*_*a*_ is the number of atoms, and Δ is the detuning of write (read) light 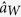 (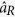) and Stokes (anti-Stokes) light 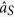 (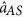). Here, we have supposed that all detuning is the same without the loss of generality.

At first, the atoms are prepared in ground state |*g*〉 by an optical pump field. When a strong write pulse is applied onto the atoms, the SRS process happens and the interaction Hamiltonian is similar to that of an optical parametric down conversion process^20^, i.e.


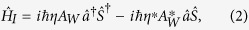


where the strong write pulse has been treated as a classical light and its normalized amplitude *A*_*W*_ is proportional to the Rabi frequency Ω_*W*_ of the write optical field.

According to the Heisenberg motion equation 
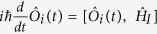
, we can get the dynamic equation of above-mentioned operators. By solving the Heisenberg equations of the light and atomic spin wave, we obtain the solutions of time-dependent operators:


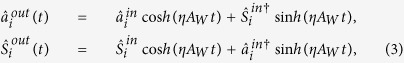


here *i* = 1 and 2 correspond to the light-atom entanglement produced in atomic ensembles A and B, respectively. According to the basis of operator linearization, in which operators can be taken into account the sum of the mean value 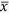 (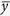) and the fluctuations 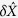 (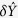), 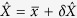, 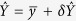. The input optical fields 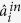 are considered as a vacuum state, and the fluctuations of them are normalized: 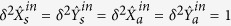. The inseparability criterion proposed by Duan and Simon is the sufficient condition of entanglement between two optical beams, which has been extended to verify the entanglement between the light and atoms^30^,^31^. If the sum of correlation variances between light (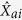, 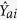) and atoms (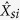, 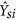) quadratures is less than 4, the entanglement of light and atoms exists. The correlation variance V of the light and atoms equals to:


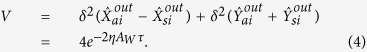


*τ* is the time of interaction between light and atoms. It is obviously that the correlation degree of light and atoms depends on the correlation parameter:


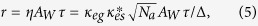


where r stands for the correlation parameter. The larger the *r* is, the lower the quantum correlation is. As *r* = 0, *V* = 4 corresponds to that of a coherent state which is defined as the quantum noise limit (QNL). When *r* > 0, the correlation variance V will be less than QNL, which means that the entanglement between light and atoms exists. When *r* → ∞, we have *V* → 0 and the perfect correlation is obtained.

### Entangling two atomic ensembles via entanglement swapping

In the following we consider how to obtain the entanglement of atomic ensembles A and B. Two Stokes optical fields 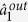 and 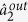 respectively generated by atomic ensembles A and B via SRS process are coupled on a 50/50 optical beam splitter with a phase difference of 0 at node C, and the output interference optical fields 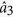 and 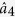 are written as:


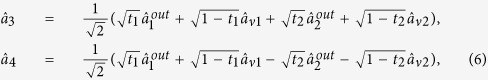


where *t*_1_ (*t*_2_) is the imperfect transmission efficiency of 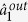 (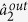), and 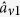 (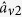) is the vacuum noise caused by the transmission losses.

Then the interference signals 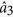 and 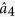 are detected by BHD1 and BHD2, respectively. When the phase difference between 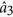 (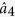) and the local oscillator 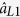 (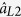) is locked on *π*/2 (0), the quantum fluctuation of the phase (amplitude) quadrature of 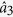 (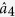) will be measured^32^. The fluctuation 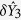 of the phase quadrature of the optical field 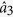 and the fluctuation 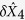 of the amplitude quadrature of optical field 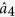 are expressed by:


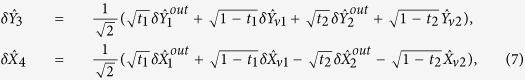


where 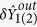 and 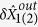 are the quantum fluctuations of the phase and the amplitude quadrature measured by BHD1 (BHD2), respectively, as well as 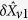 (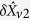) and 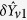 (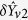) are quadrature quantum fluctuations of the above vacuum noise.

Finally, the measured signals 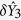 and 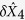 are fed back to the collective atomic spin field 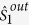 of the atomic ensemble A through a classical channel composed of a radio frequency coil with tunable normalized classical gain factors *g*_1_ and *g*_2_ for the fed-back signals of the amplitude and phase quadratures, respectively^33^. The atomic state of atomic ensemble A will involve the quantum information of 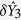 and 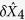, that is:


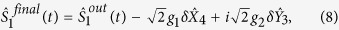


where 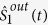 is the resultant collective atomic spin field of the atomic ensemble A after the interference of 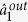 and 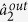, and 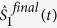 is the final collective atomic spin field after receiving the fedback signals. By choosing proper classical gain factors, the optimal correlation of atomic ensembles can be achieved. Usually and without the loss of generality, we take *g*_1_ = *g*_2_ = *g*, and *t*_1_ = *t*_2_ = *t*_0_.

From equation (3) and equation (8) the correlation variance V^'^ of the two atomic ensembles is obtained:


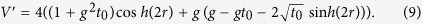


The transmission losses for light is unavoidable in practical systems, which influence the entanglement swapping quality.

The entanglement swapping quality is also limited by the atomic coherence lifetime. The time to maintain the entanglement is determined by the atomic coherence lifetime^28^. The atomic coherence lifetime, which dominates the decay process of entanglement, is usually more than 5 ms in hot atomic ensembles^26^, and can be up to 40 ms^29^. The period of the real-time measurement and feedback for swapping operation is much shorter than the atomic coherence lifetime, and therefore the coherence lifetime is long enough to build the entanglement of two atomic ensembles.

By calculating the minimal value of equation (9) versus *g*, we get^7^:


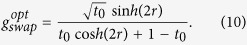


When the optimal gain factor is chosen, the best entanglement will be obtained. The optimal gain factor depends on the correlation parameter r.

### Characterizing atom-atom entanglement

**Figure 3:**
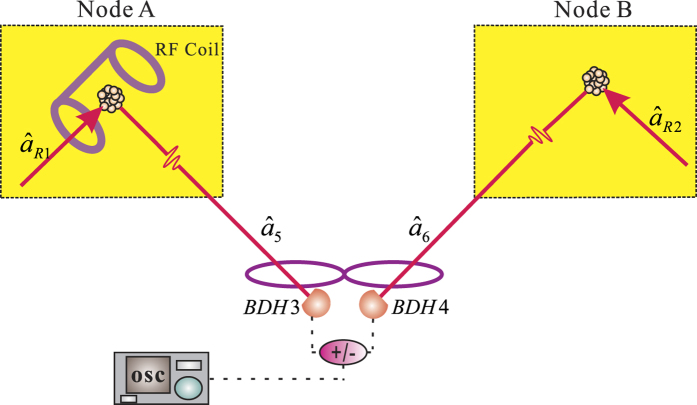
Schematic for verifying atom-atom entanglement. BHD3 (4): balanced homodyne detector; RF Coil: radio frequency coil; +/−: positive/negative power combiner; OSC: oscilloscope.

For verifying and characterizing the entanglement of two atomic ensembles, we respectively apply read optical pulses 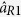 and 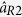 onto ensembles A and B at the same time, to convert the atomic quantum state into quantum state of the anti-Stokes light via SRS process, as shown in Fig. 3. The correlation variances of two anti-Stokes optical beams respectively coming from the two ensembles will characterize the entanglement between the two atomic ensembles.

**Figure 4:**
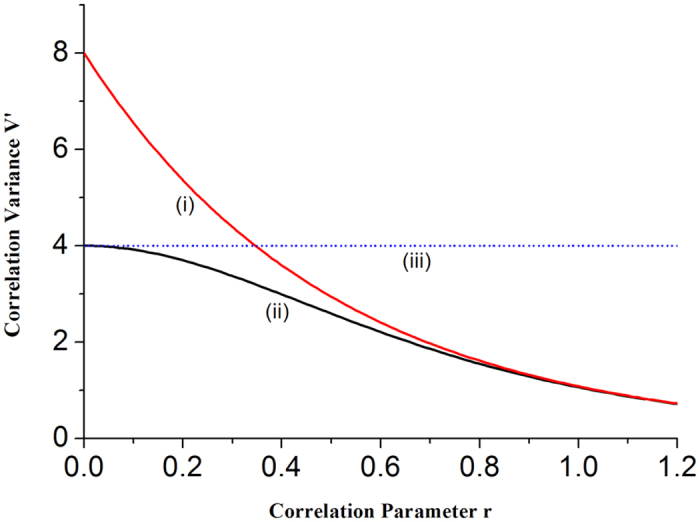
Dependence of the correlation variances on the correlation parameter *r*. Trace (i): the correlation variance with *g* = 1; trace (ii): the correlation variance with 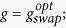 and trace (iii): QNL.

We analyze the dependence of the correlation variances on the correlation parameter *r* in Fig. 4. Trace (i) and trace (ii) correspond to *g* = 1 and 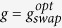, respectively, and trace (iii) is QNL. It can be seen that when the correlation parameter *r* increases, the interaction between light and atoms is strengthened, thus the correlation variance V′ reduce. When *V*′ < 4 (QNL), the two atomic ensembles are entangled. For *g* = 1, when r is less than 0.35, the atom-atom entanglement exists. However, if the optimal gain is used, the atom-atom entanglement will always be created for any nonzero *r* values. For smaller *r* values the optimal gain plays a significant role to produce the entanglement. When *r* > 0.8 two curves overlap and the values of the gain factor are no longer important. In present experimental systems the correlation parameter *r* is smaller than 0.8^34^,^35^,^36^, therefore the optimal gain factor should be applied to obtain better atom-atom entanglement.

**Figure 5:**
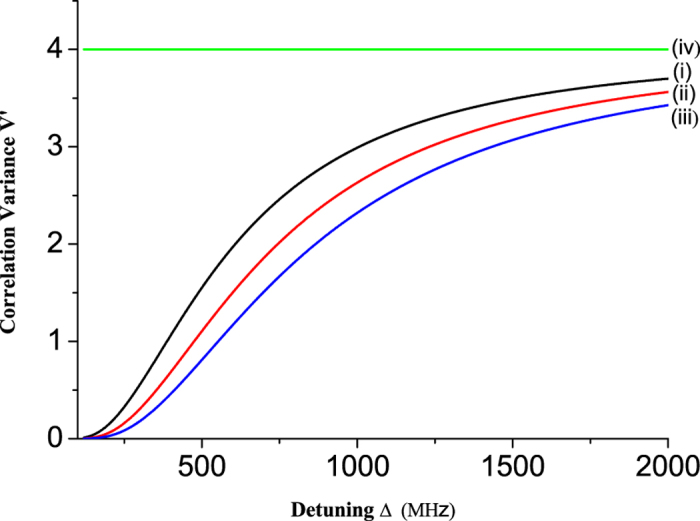
Functions of the correlation variances on the detuning of light and atoms interaction. Trace (i), (ii) and (iii) correspond the correlation variances on the detuning of light and atoms interaction when Ω_*W*_ = 5 MHz, 6 MHz and 7 MHz, respectively. Trace (iv) represents QNL.

The function of the correlation variances versus the detuning Δ is illustrated in Fig. 5, when 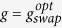 is taken. The trace (i) to (iii) correspond to the Rabi frequency Ω_*W*_ = 5 MHz, 6 MHz and 7 MHz, respectively, and trace (iv) is QNL. For a given detuning Δ, the correlation variances decrease when the Rabi frequency of the write optical pulse Ω_*W*_ increase. From Fig. 5, we can see that the atom-atom entanglement reaches the best value for zero detuning in ideal condition. However, in the real experiment the harmful extra noise increased, which will reduce the entanglement, if the detuning is too small, and thus the scheme has to work at a certain detuning^34^,^37^. The numerical calculation show that −4.3 dB entanglement between two atomic ensembles is obtained in a 700 MHz detuning via light-atom mixed entanglement swapping, which is better than the result of the optical entanglement swapping^7^. To avoid the huge extra noise at the atomic resonance the detuning has been applied in many experimental systems of quantum optics, such as, Appel J. *et al.*^34^ illustrate that 630 MHz is the optimal detuning for quantum memory of squeezed light in Rb atomic ensemble by means of EIT approach^34^; Qin Z.Z. *et al.*^37^ demonstrate that 800 MHz detuning is the best choice, and −7 dB intensity-difference squeezing in Rb atomic ensemble based on four-wave mixing is experimentally generated^37^. Thus in the systems of light-atom interaction we have to take an appropriate compromising between high efficiency and large noise by using a certain detuning.

## Discussion

We have proposed a scheme to deterministically establish the CV entanglement between two distant macroscopic atomic ensembles via light-atom mixed entanglement swapping. The entanglement of light and atoms is generated by means of the SRS process. The entanglement of two distant atomic ensembles is achieved by the interference of two optical beams coming from two atomic ensembles respectively and the feedback of the measured resultants. The dependence of the atom-atom entanglement on the parameters of the system is numerically calculated. The proposed scheme of building atom-atom entanglement has potential applications in future quantum information networks for realizing the entanglement among remote nodes.

## Additional Information

**How to cite this article**: Liu, Y. *et al.* Deterministically Entangling Two Remote Atomic Ensembles via Light-Atom Mixed Entanglement Swapping. *Sci. Rep.* **6**, 25715; doi: 10.1038/srep25715 (2016).
